# Frailty transitions and cognitive function among South Korean older adults

**DOI:** 10.1038/s41598-021-90125-6

**Published:** 2021-05-20

**Authors:** Fatima Nari, Bich Na Jang, Hin Moi Youn, Wonjeong Jeong, Sung-In Jang, Eun-Cheol Park

**Affiliations:** 1 Department of Public Health, Graduate School, Yonsei University, Seoul, Republic of Korea; 2 Institute of Health Services Research, Yonsei University, Seoul, Republic of Korea; 3 Department of Preventive Medicine, Yonsei University College of Medicine, 50 Yonsei-ro, Seodaemun-gu, Seoul, 03722 Republic of Korea

**Keywords:** Neuroscience, Risk factors

## Abstract

Frailty is considered a multidimensional geriatric syndrome, manifested by the accumulation of age-associated deficits. The consequences of frailty transitions are still understudied. This study evaluated the influence of frailty transitions on cognitive function in the older adult population. We used data derived from the Korean Longitudinal Study of Aging (KLoSA) (2008–2018) on older adults aged ≥ 65 years. Frailty was assessed using a validated Korean frailty measure known as the frailty instrument (FI), and cognitive function was measured using the Korean version of the Mini-Mental State Examination (K-MMSE). Transitions in frailty and their relationship with cognitive function were investigated using lagged generalized estimating equations (GEE), t-tests, and ANOVA. Respondents who experienced frailty transitions (those with ameliorating frailty), those who developed frailty, and whose frailty remained constant, were more likely to have a lower cognitive function than those who were consistently non-frail. Older age, activities of daily living (ADL) disability, and instrumental ADL disability were more negatively associated with declining cognitive function, especially in the “frail → frail” group. Changes in all individual components of the frailty instrument were significantly associated with impaired cognitive function. The results suggest an association between frailty transitions and cognitive impairment. Over a 2-year span, the remaining frail individuals had the highest rate of cognitive decline in men, while the change from non-frail to frail state in women was significantly associated with the lowest cognitive function values. We recommend early interventions and prevention strategies in older adults to help ameliorate or slow down both frailty and cognitive function decline.

## Introduction

Frailty has been widely recognized as a geriatric syndrome and an emerging risk factor for adverse health outcomes in older adults. Frailty in older adults is linked to a greater risk of falls, hospitalization, disability, and death^1,2^. In Korea, reports indicate that the prevalence of frailty ranges from 2.5 to 31.7%, depending on the study sample and frailty scale measure^3–5^. Considering the significance and impact of frailty in the aging population, interest in and research regarding frailty has gradually increased^6^.

Since frailty is a broad concept with various known stressors and causative factors, it may present differently in various populations of interest^7^. This has led to the development of numerous instruments and scales to measure frailty in the aging demographic^8,9^. However, salient features of frailty seem to be common, with self-reported exhaustion and grip strength weakness recurring in its definition^10^.

One of the earliest definitions by Fried et al. looked at the conceptualization of frailty in a unidimensional manner by considering only the physical aspect of frailty^7^. However, more recent studies have begun to define frailty as a multidimensional measure with various age-associated deficits (e.g., physical, affective, social, etc.). For this reason, we employed the frailty instrument (FI), a frailty measure developed and validated in the Korean population for rapid assessment of frailty and determination of negative health outcomes in older adults^3,11^. The FI encompassed a wider frailty approach, including items in the physical, psychological, and social domain^5^.

Prior studies have shown that frailty is dynamic, demonstrating the changeability of frailty over time^12,13^. Transitions in frailty status may be bidirectional, with improvement or worsening of initial frailty, and could be related to the complex and multidimensional nature of frailty. To date, little is known about the dynamics of frailty transition over time. One study used the CHS frailty criteria to explore 18-month frailty transitions in older adults and revealed that worsening of frailty was more common (rates up to 43.3%) than improvement in frailty (rates up to 23.0%)^14^.

Moreover, despite some longitudinal studies incorporating the dynamic nature of frailty, the majority have focused on the predictive factors of frailty transitions^15,16^, while the outcomes of such frailty changes are still understudied.

Cross-sectional studies have suggested that the physical phenotype of frailty and cognitive impairment share common biological pathways, with associations between the two mechanisms being reported in various populations^17–20^ and Korea as well^21^. Cognitive impairment and dementia are significant public health consequences in the aging demographic^17^. Therefore, numerous research efforts have been put forth towards preventing, slowing, and ameliorating cognitive decline.

Nevertheless, little is known about the influence of wider conceptualizations of frailty and its transitions on cognitive impairment. Therefore, based on these considerations, we attempted to investigate the effect of frailty transitions on cognitive function among older adults in South Korea using a broader model of frailty.

## Results

Table 1 lists the baseline characteristics of the 2375 respondents (2008 → 2010). The median K-MMSE score in men was highest for the Non-frail → non-frail group (27.0 [IQR 4.0], 25.0 [IQR 6.0] for the Frail → non-frail group, 24.0 [IQR 7.5] for the Non-frail → frail group, 21.0 [IQR 8.0] for the frail → frail group). Similarly, in women, the non-frail → non-frail group had the highest median K-MMSE score 25.0 [IQR 6.0], 22.0 [IQR 9.0] for the frail → non-frail group, 21.0 [IQR 8.0] for the non-frail → frail group, and 18.0 [IQR 8.5] for the frail → frail group.

**Table 1:** General characteristics of study population at baseline (N = 2375).

Variables	Cognitive function (K-MMSE)									
	Men					Women				
	N	(%)	Median	IQR	P-value	N	(%)	Median	IQR	P-value
**Total**	1101	(100.0)	27.0	5.0	< 0.0001	1274	(100.0)	24.0	8.0	< 0.0001
**Frailty transitions (2008 → 2010)**					< 0.0001					< 0.0001
Non-frail → non-frail	815	(74.0)	27.0	4.0		798	(62.6)	25.0	6.0	
Frail → non-frail	79	(7.2)	25.0	6.0		130	(10.2)	22.0	9.0	
Non-Frail → frail	128	(11.6)	24.0	7.5		174	(13.7)	21.0	8.0	
Frail → frail	79	(7.2)	21.0	8.0		172	(13.5)	18.0	8.5	
**Age**					< 0.0001					< 0.0001
65–74	678	(61.6)	27.0	5.0		757	(59.4)	25.0	7.0	
75–84	372	(33.8)	26.0	6.0		446	(35.0)	22.0	9.0	
85 ≤	51	(4.6)	24.0	10.0		71	(5.6)	17.0	11.0	
**Education level**					< 0.0001					< 0.0001
Lower than middle school	523	(47.5)	25.0	6.0		1,065	(83.6)	23.0	9.0	
Middle school graduate	184	(16.7)	27.0	4.0		118	(9.3)	26.0	6.0	
High School graduate	261	(23.7)	27.0	4.0		76	(6.0)	27.0	6.0	
University graduate	133	(12.1)	28.0	4.0		15	(1.2)	28.0	6.0	
**Income level**					0.0004					< 0.0001
Low	486	(44.1)	26.0	6.0		664	(52.1)	23.0	7.0	
Middle Low	312	(28.3)	27.0	5.0		278	(21.8)	25.0	7.0	
Middle High	200	(18.2)	27.0	5.0		192	(15.1)	24.0	10.0	
High	103	(9.4)	28.0	5.0		140	(11.0)	23.5	9.0	
**Marital status**					0.0026					< 0.0001
Married	986	(89.6)	27.0	5.0		616	(48.4)	24.0	7.0	
Unmarried	115	(10.4)	25.0	7.0		658	(51.6)	23.0	10.0	
**Economic activity**					< 0.0001		(0.0)			0.1967
Active	402	(36.5)	28.0	4.0		223	(17.5)	24.0	7.0	
Inactive	699	(63.5)	26.0	5.0		1051	(82.5)	23.0	8.0	
**Region**					0.0316					0.0052
Urban	740	(67.2)	27.0	5.0		868	(68.1)	24.0	8.0	
Rural	361	(32.8)	26.0	7.0		406	(31.9)	23.0	9.0	
**Smoking**					0.5593					0.0781
Current	311	(28.2)	26.0	6.0		33	(2.6)	22.0	8.0	
Past	379	(34.4)	27.0	5.0		24	(1.9)	24.0	8.5	
Never	411	(37.3)	27.0	5.0		1217	(95.5)	24.0	8.0	
**Drinking**					0.0423					0.6524
Current	532	(48.3)	27.0	5.0		137	(10.8)	23.0	7.0	
Past	289	(26.2)	26.0	5.0		60	(4.7)	22.5	7.0	
Never	280	(25.4)	26.0	6.0		1077	(84.5)	24.0	8.0	
**BMI**					0.6466					0.0001
Overweight	180	(16.3)	26.0	5.5		326	(25.6)	25.0	7.0	
Normal	845	(76.7)	27.0	5.0		871	(68.4)	23.0	8.0	
Underweight	76	(6.9)	27.0	6.0		77	(6.0)	21.0	8.0	
**Regular physical activity**					0.0031					< 0.0001
Yes	383	(34.8)	27.0	4.0		253	(19.9)	26.0	6.0	
No	718	(65.2)	26.0	6.0		1021	(80.1)	23.0	9.0	
**ADL limitations**					0.0012					0.0037
Yes	23	(2.1)	20.0	12.0		42	(3.3)	18.5	12.0	
No	1078	(97.9)	27.0	5.0		1232	(96.7)	24.0	8.0	
**IADL limitations**					0.0001					< 0.0001
Yes	144	(13.1)	25.0	6.5		156	(12.2)	18.0	9.0	
No	957	(86.9)	27.0	5.0		1118	(87.8)	24.0	8.0	
**Number of chronic diseases**					0.2799					0.6590
0	446	(40.5)	27.0	5.0		462	(36.3)	24.0	9.0	
1	402	(36.5)	27.0	5.0		518	(40.7)	23.0	8.0	
≥ 2	253	(23.0)	26.0	5.0		294	(23.1)	23.0	8.0	
**Lagged dependent variable**										
MMSE score of prior year	1101	(100.0)	27.0	5.0	< 0.0001	1274	(100.0)	24.0	7.0	< 0.0001

Table 2 shows the adjusted effects of transitions in frailty on cognitive function. Compared to those in the non-frail → non-frail group, for the frail → non-frail group, the estimate was β = − 0.628 (p = 0.013), while for those in the non-frail → frail group, the estimate was β = − 1.635 (p < 0.0001), and for those in frail → frail group, the estimate was β = − 1.879 (p < 0.0001). In women, compared to the non-frail → non-frail group, the lowest estimate was in the frail → non-frail group β = − 0.276 (p = 0.203), in the Non-frail → frail group, β = − 1.799 (p < 0.0001), and β = − 1.651 (p < 0.0001) in the frail → frail group.

**Table 2:** Association of frailty transitions and cognitive function.

Variables	Cognitive function (K-MMSE)					
	Men			Women		
	ß	SE	P-value	ß	SE	P-value
**Frailty transitions**						
Non-frail → non-frail	**Ref.**			**Ref.**		
Frail → non-frail	− 0.628	0.254	0.013	− 0.276	0.217	0.203
Non-frail → frail	− 1.635	0.222	< 0.0001	− 1.799	0.215	< 0.0001
Frail → frail	− 1.879	0.252	< 0.0001	− 1.651	0.216	< 0.0001
**Age**						
65–74	**Ref.**			**Ref.**		
75–84	− 0.298	0.103	0.004	− 0.545	0.110	< 0.0001
85 ≤	− 0.789	0.230	0.001	− 1.449	0.243	< 0.0001
**Education level**						
Lower than middle school	− 0.769	0.152	< 0.0001	− 1.121	0.192	< 0.0001
Middle school graduate	− 0.480	0.164	0.003	− 0.569	0.229	0.013
High School graduate	− 0.351	0.141	0.013	− 0.782	0.250	0.002
University graduate	**Ref.**			**Ref.**		
**Income level**						
Low	0.253	0.189	0.181	− 0.173	0.193	0.370
Middle low	0.350	0.190	0.066	0.081	0.200	0.688
Middle high	0.389	0.201	0.053	− 0.009	0.221	0.966
High	**Ref.**			**Ref.**		
**Marital status**						
Married	**Ref.**			**Ref.**		
Unmarried	− 0.126	0.174	0.470	− 0.161	0.110	0.145
**Economic activity**						
Active	**Ref.**			**Ref.**		
Inactive	− 0.564	0.114	< 0.0001	− 0.306	0.149	0.040
**Region**						
Urban	0.237	0.122	0.052	0.431	0.124	0.001
Rural	**Ref.**			**Ref.**		
**Smoking**						
Current	0.060	0.144	0.676	− 0.456	0.477	0.339
Past	− 0.063	0.119	0.596	0.058	0.268	0.828
Never	**Ref.**			**Ref.**		
**Drinking**						
Current	0.015	0.129	0.905	0.178	0.156	0.253
Past	− 0.160	0.150	0.287	− 0.415	0.228	0.069
Never	**Ref.**			**Ref.**		
**BMI**						
Overweight	− 0.086	0.133	0.516	0.406	0.124	0.001
Normal	**Ref.**			**Ref.**		
Underweight	0.252	0.222	0.256	− 0.194	0.251	0.441
**Regular physical activity**						
Yes	**Ref.**			**Ref.**		
No	− 0.459	0.107	< 0.0001	− 0.474	0.124	0.000
**ADL limitations**						
Yes	− 3.896	0.708	< 0.0001	0.167	0.481	0.729
No	**Ref.**			**Ref.**		
**IADL limitations**						
Yes	− 0.670	0.195	0.001	− 1.617	0.249	< 0.0001
No	**Ref.**			**Ref.**		
**Number of chronic diseases**						
0	**Ref.**			**Ref.**		
1	0.256	0.110	0.019	− 0.208	0.128	0.104
≥ 2	− 0.005	0.133	0.969	− 0.385	0.151	0.011
**Lagged dependent variable**						
MMSE score of prior year	0.600	0.018	< 0.0001	0.665	0.014	< 0.0001

*K-MMSE* Korean-Mini Mental Status Evaluation, *β* regression coefficient, *SE* standard error.

Table 3 shows the subgroup analysis for age, ADL limitations, IADL limitations, and the number of chronic diseases associated with frailty transitions and cognitive function. For both men and women, the frail → frail group in particular had the lowest K-MMSE estimate for respondents aged ≥ 85 years; β = − 2.318 (p = 0.0002), β = − 2.838 (p < 0.0001) than their younger counterparts. Subgroup analysis for ADL limitations did not show statistical significance for men; however, women with ADL limitations in the non-frail → frail group; β = − 4.956 (p < 0.0001) and frail → frail group; β = − 4.025 (p = 0.001) showed statistically significant lower K-MMSE estimates. Subgroup analysis for men who experienced IADL limitations tended to show lower estimates across all frailty transition groups: frail → non-frail; β = − 1.090 (p = 0.253), Non-Frail → Frail; β = − 2.166 (p = 0.001) and frail → frail; β = − 2.120 (p = 0.005). IADL limitations in women showed statistically significant lower estimates in the non-frail → frail group; β = − 2.569 (p < 0.0001) and the frail → frail group; β =  − 1.546 (p = 0.008). Multimorbidity did not show a dose–response relationship in our study. Those in the frail, → frail group with no chronic diseases in men had a lower cognitive function estimate, β = − 2.547 (p < 0.0001), and 1 chronic disease in women; β = 1.938 (p < 0.0001), respectively.

**Table 3:** The results of subgroup analysis stratified by covariates.

Variables	Cognitive function (K-MMSE)											
	Frailty transitions											
	Non-frail → non-frail			Frail → non-frail			Non-frail → frail			Frail → frail		
	ß	SE	P-value	ß	SE	P-value	ß	SE	P-value	ß	SE	P-value
**Men**												
Age												
65–74	**Ref.**			− 1.083	0.457	0.0177	− 1.401	0.395	0.000	− 1.480	0.482	0.002
75–84	**Ref.**			− 0.859	0.345	0.013	− 1.692	0.281	< 0.0001	− 2.141	0.371	< 0.0001
85 ≤	**Ref.**			0.661	0.869	0.4470	− 1.496	0.644	0.020	− 2.318	0.626	0.000
ADL limitations												
Yes	**Ref.**			2.585	2.134	0.226	− 3.678	2.068	0.075	− 0.615	2.072	0.767
No	**Ref.**			− 0.732	0.256	0.0042	− 1.542	0.214	< 0.0001	− 1.870	0.246	< 0.0001
IADL limitations												
Yes	**Ref.**			− 1.090	0.953	0.253	− 2.166	0.660	0.001	− 2.120	0.751	0.005
No	**Ref.**			− 0.673	0.250	0.0072	− 1.552	0.224	< 0.0001	− 1.893	0.259	< 0.0001
Number of chronic diseases												
0	**Ref.**			− 0.822	0.418	0.0493	− 1.466	0.373	< 0.0001	− 2.547	0.509	< 0.0001
1	**Ref.**			− 1.041	0.391	0.008	− 1.760	0.343	< 0.0001	− 2.207	0.428	< 0.0001
≥ 2	**Ref.**			0.037	0.549	0.9467	− 1.680	0.415	< 0.0001	− 1.599	0.370	< 0.0001
**Women**												
Age												
65–74	**Ref.**			− 0.587	0.352	0.0958	− 1.343	0.383	0.001	− 1.807	0.387	< 0.0001
75–84	**Ref.**			− 0.412	0.293	0.160	− 2.159	0.287	< 0.0001	− 1.795	0.291	< 0.0001
85 ≤	**Ref.**			0.251	0.752	0.7382	− 2.569	0.628	< 0.0001	− 2.838	0.555	< 0.0001
ADL limitations												
Yes	**Ref.**			1.381	1.494	0.355	− 4.956	1.163	< 0.0001	− 4.025	1.216	0.0001
No	**Ref.**			− 0.106	0.309	0.7328	− 1.163	0.300	0.000	− 1.479	0.243	< 0.0001
IADL limitations												
Yes	**Ref.**			0.182	0.725	0.802	− 2.569	0.582	< 0.0001	− 1.546	0.578	0.008
No	**Ref.**			− 0.428	0.225	0.0570	− 1.655	0.226	< 0.0001	− 1.851	0.229	< 0.0001
Number of chronic diseases												
0	**Ref.**			− 1.131	0.388	0.0036	− 1.628	0.418	< 0.0001	− 1.817	0.474	0.000
1	**Ref.**			− 0.274	0.351	0.435	− 1.829	0.323	< 0.0001	− 1.938	0.300	< 0.0001
≥ 2	**Ref.**			0.092	0.375	0.8056	− 2.015	0.375	< 0.0001	− 1.505	0.400	0.000

*K-MMSE* Korean-Mini Mental Status Evaluation, *β* regression coefficient, *SE* standard error.

Table 4 shows the subgroup analysis of changes in each component of the frailty instrument with cognitive function. Compared to the no → no group, the yes → yes group showed the lowest estimates in men for change in exhaustion: β = − 1.665 (p < 0.0001), social isolation; β = − 1.211 (p < 0.0001), and weakness of grip strength; β = − 1.226 (p < 0.0001), respectively. On the other hand, compared to the no → no group in women, the no → yes group showed the lowest K-MMSE estimates for change in exhaustion: β = − 1.370 (p < 0.0001) and weakness of grip strength; β =  − 1.358 (p < 0.0001), while for social isolation, yes → yes group β = − 1.059 (p < 0.0001).

**Table 4:** Subgroup analysis of Frailty Instrument (FI) components with cognitive function.

Variables		Cognitive function (K-MMSE)					
		Men			Women		
		ß	SE	P-value	ß	SE	P-value
**Change in exhaustion**							
No → no		**Ref.**			**Ref.**		
Yes → no		− 0.142	0.201	0.480	− 0.148	0.191	0.438
No → yes		− 0.868	0.192	< 0.0001	− 1.370	0.196	< 0.0001
Yes → yes		− 1.665	0.283	< 0.0001	− 1.289	0.215	< 0.0001
**Change in social isolation**							
No → no		**Ref.**			**Ref.**		
Yes → no		− 0.131	0.206	0.524	0.057	0.205	0.783
No → yes		− 0.965	0.191	< 0.0001	− 1.035	0.195	< 0.0001
Yes → yes		− 1.211	0.176	< 0.0001	− 1.059	0.154	< 0.0001
**Change in weakness of grip strength**							
No → no		**Ref.**			**Ref.**		
Yes → no		− 0.268	0.176	0.127	− 0.017	0.191	0.931
No → yes		− 0.751	0.161	< 0.0001	− 1.358	0.185	< 0.0001
Yes → yes		− 1.226	0.188	< 0.0001	− 0.877	0.171	< 0.0001

*K-MMSE* Korean-Mini Mental Status Evaluation, *β* regression coefficient, *SE* standard error.

## Discussion

Through our findings, we confirmed that respondents aged 65 years and above, who experienced increased frailty, had significantly lower cognitive function than those who were continuously non-frail. Our results also showed that while ameliorating frailty reduced cognitive function impairment in individuals, compared to those who became frail or who remained frail over a 2-year interval, they still exhibited cognitive decline compared to non-frail individuals. Moreover, our primary analysis results revealed that while the frail → frail group showed the lowest MMSE estimate in men, women in the non-frail → frail group presented the lowest estimates. The sensitivity analysis results comparing transitions between robust, prefrail, and frail states and cognitive function (Supplementary Table 1) supported our primary findings as well; men had the lowest MMSE estimates in the frail → frail group. In contrast, women in the robust → frail group showed significantly lower cognitive function estimates. Furthermore, the prevalence of differences in FI scores (Supplementary Table 2) revealed that men and women with unchanging scores over 2 years made up the largest prevalence (approximately 50%), followed by 1-point transitions in either direction. Both sexes were least likely to experience 3-point transitions, followed by 2-point transitions.

Comparing our study’s findings has proved to be difficult because of differences in the definition of frailty in prior literature. Numerous observational studies have shown a chronological relationship between frailty and cognitive impairment^6^. Prior evidence suggests that the pathways linked with frailty in older adults are similar to those that promote neurodegeneration, including chronic inflammation and oxidative stress and consequent cognitive decline^22–24^. A cohort study reported that physical frailty among older persons with no cognitive impairment at baseline was associated with an increased risk of the development of mild cognitive impairment during 12 years of follow-up^25^. A longitudinal study by Chong et al. reported that 1-year frailty transitions measured using modified CHS criteria in older adults with cognitive impairment were not significantly associated with cognitive decline in earlier cognitive impairment stages but became significant in patients with mild to moderate Alzheimer’s disease^26^. Furthermore, the study suggested the need for further studies with larger samples and extended follow-up time^26^.

The relationship between older age, frailty, and cognitive impairment has long been postulated in previous studies. The highest cognitive decline in the oldest age group (≥ 85) years in our study is an expected finding supported by other studies since both frailty, and cognitive impairment are age-related syndromes^18^. The proposed pathophysiological mechanisms behind this phenomenon are numerous, suggesting that with aging, there is an accumulation of DNA damage due to apoptosis, mitochondrial dysfunction, and increased synthesis of reactive oxygen species, leading to oxidation and damage to proteins and lipids in the brain^17,27^.

Moreover, pertaining to the two other variables investigated in our subgroup analysis, ADL and IADL disabilities were associated with lowered cognitive function in individuals experiencing frailty transitions. However, our study’s results did not show a significant relationship with the number of comorbidities and cognition. Several studies have suggested that frailty, number of chronic diseases, and disability are closely related concepts^13^. In the CHS study^7^, the prevalence of physical frailty, comorbidity, and disability^28^ was investigated. The coexistence of all three variables was found in 21.5% of the study sample. However, frail participants, free from any comorbidity or disability, occurred in 26.6% of the study group, indicating that frailty is an independent factor different from comorbidity and disability. It has been revealed that older persons frequently transition between states of disability and independence^29^. Another study found an association between physical frailty, cognition, and limitations in IADL in older persons^30^. Conversely, another study found that frail participants with cognitive impairment were more likely to have ADL or IADL disabilities^31^. Contrary to our study’s results, chronic diseases have been identified as a risk factor for both frailty and cognitive impairment, suggesting that all three factors share a common pathway of inflammation^18,32^.

Our study’s subgroup analysis of our variable of interest also showed that the unfavorable transitions in individual components of the FI are negatively associated with the impacted cognitive function. Although prior studies have stated that grip strength is the most important predictor of cognitive decline among all frailty domains, our results showed otherwise^25,33,34^. Self-reported exhaustion revealed the lowest K-MMSE scores in both sexes, despite some studies reporting that it did not significantly affect cognitive function^25^. Nevertheless, Ma et al. reported that exhaustion is significantly associated with poor global cognition^35^. Additionally, continuous social isolation is confirmed to have a detrimental effect on cognitive function. Participation in social activities has also been shown to have a protective effect against cognitive impairment and is linked to better memory by increasing brain volume and improving cognition^36,37^.

This study has several strengths and limitations. Regarding the methodological implications of our study, to our knowledge, only a few longitudinal studies have documented an association between changes in frailty over time in older people and cognitive function. Thus, exploring the dynamics of change over time of frailty on cognitive function provides novel information compared to previous studies. Another strength is that the sample drawn for our study is representative of the overall population. Our study’s sample can be generalized to South Korean older adults aged 65 years or older.

Nevertheless, this study has several limitations. Although the frailty instrument has been previously developed and validated in the Korean population, the measure of frailty used in this study is relatively simple and not a universally used scale. Second, although we analyzed our study’s data longitudinally, we could not reverse causality between frailty transitions and cognitive function.

In conclusion, our findings suggest that 2-year frailty transitions negatively affect cognitive function in older adults in South Korea. Our study provides longitudinal evidence to the growing body of literature that proposes that frailty and cognitive decline share common pathways and risk factors.

Moreover, cognitive impairment was associated not only with constant or developing frailty but also with ameliorating frailty. Based on these results, early intervention and prevention strategies at the physical, nutritional, and social levels are recommended to best tackle frailty and cognitive impairment issues in older adults.

## Methods

**Figure 1:**
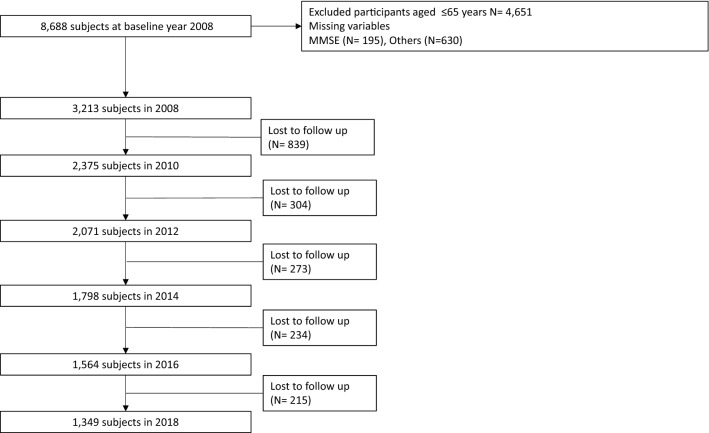
Flowchart of the study participants from 2008 to 2018.

Our present study extracted data over 10 years from the 2nd to 7th wave (2008 to 2018) of the Korean Longitudinal Study of Aging (KLoSA). Since its conception in 2006, the Korea Labor Institute has been collecting routine panel data in the same population sample of older residents aged more than 45 years from all regions around Korea, apart from Jeju Island. The total number of participants included in 2008 was 8875 (approximately 84.7% of the original 10,254 participants in 2006). The survey was conducted biennially, with a sample retention rate of 77.7% in 2018. More information about the survey can be found on the panel survey organization website (https://survey.keis.or.kr/eng/klosa/klosa01.jsp). The exclusion criteria included age under 65, missing information on variables, and upon follow-up, leading to the inclusion of 3213 participants in 2008, 2375 in 2010, 2071 in 2012, 1798 in 2014, 1564 in 2016, and 1349 participants in 2018. The flow of participants and the selection process are shown in detail in Fig. 1.

### Measurement of cognitive function

Respondents’ cognitive function was measured using the Korean version of the Mini-Mental State Examination (K-MMSE) score. The K-MMSE is a validated construct used to evaluate cognitive functioning in the Korean population^38^. The instrument consists of seven cognitive function categories: time orientation, spatial orientation, registration, attention and calculation, recall, language, and visual construction domains. These items comprise a composite score of 30 points, with higher scores indicating higher cognitive function.

### Frailty transition assessment

Transitions in frailty status were assessed by measuring changes in frailty criteria defined by the frailty instrument (FI). The FI was developed and validated in the Korean older adult population for the rapid assessment of frailty and linked adverse outcomes such as disability, institutionalization and mortality, demonstrating high predictive validity, discrimination and calibration power^3,11^. The FI captures the social, psychological, and physical phenotypes of frailty by incorporating three criteria: exhaustion, social isolation, and weakness of grip strength. The exhaustion criterion was assessed using self-reported measures of individuals feeling that any task required effort or that they could not get going the preceding week. Social isolation was established if respondents reported not participating in any social group activity. Weakness was calculated using sex-specific grip strength: < 24 kg for men and < 15 kg for women. The three variables were used to compute a score of 3 points, with ≥ 2 classified as frail, ≥ 1 classified as pre-frail, and ≤ 1 as robust. We dichotomized frailty status into frail (≥ 2) and Non-Frail (≤ 1) for our study's purpose. The lag function was applied to detect changes in frailty in the prior year and the succeeding year, following a 2-year gap. Thus, frailty transitions were categorized into four groups: (1) Non-frail → non-frail, (2) frail → non-frail, (3) non-frail → frail, and (4) frail → frail.

### Covariates

Data regarding sociodemographic characteristics and health-related variables were added as potential confounders in this study. Sociodemographic characteristics included sex, age (65–74, 75–84, and ≥ 85 years), educational level (middle school or lower, high school, university degree or higher), and income level per month, which was divided into four categories (low, middle-low, middle-high, and high). Additionally, marital status was classified into married and unmarried, and region (urban, rural). Economic activity was classified based on whether an individual was economically active or inactive.

Smoking and drinking status were classified as current, past, or never. BMI was classified as overweight, normal, or underweight. Physical activity was dichotomized into Yes and No, based on whether the respondents exercised regularly or not. Limitations in ADL and IADL were determined if the respondents had difficulty in performing any daily, necessary tasks (getting dressed, washing face and hands, bathing, eating meals, leaving a room, and using the toilet) for ADL, and social function related tasks (companionship and mental support, using transportation, making/receiving phone calls, managing finances, doing household chores, preparing meals, shopping, taking medications, and doing laundry). The chronic diseases included in this study were hypertension, diabetes mellitus, cancer, lung disease, heart disease, and cerebrovascular disease, and the number of comorbidities was grouped into three categories: 0, 1, and ≥ 2 diseases.

Lagged generalized estimating equation (GEE) analyses with unstructured correlation structure were conducted and controlled for confounders to provide estimates for K-MMSE scores according to the 2-year transitions in frailty. The GEE model allows for repeated measure analysis of longitudinal panel data such as the KLoSA and considers the correlation within the subject to yield the regression coefficient (β), the standard error of the coefficient (SE), and the corresponding p-value. A total of six waves (2008–2018) were used for the analysis, and repeated measurements were carried out for each individual up to five times. 2-year lagged changes in frailty were calculated using frailty status in the preceding and following years (2008–2010, 2010–2012, 2012–2014, 2014–2016, and 2016–2018) with the lag function following a 2-year interval. The GEE model was adjusted for confounding effects for all waves in our study, using the following covariates: age, educational level, income, marital status, economic activity, region, physical activity, drinking, smoking, and the number of chronic diseases. We added the lagged MMSE score as a covariate to control for cognitive function scores in the previous year. T-tests and ANOVA were applied to compare differences in respondents’ baseline traits (first time-point), and K-MMSE score distributions were summarized as the median and interquartile range (IQR). Subgroup analysis was performed to study the combined effects of frailty transition and other covariates on cognitive function. Another additional subgroup analysis was conducted to investigate the association between changes in the individual components of FI and cognitive function. Several sensitivity analyses were performed to explore the effects of frailty classification (frail, prefrail, and robust) on cognitive function, and differences in FI score changes per wave were performed. Statistical significance was set at p < 0.05. All data analyses were performed using the statistical package SAS version 9.4 (SAS Institute Inc., Cary, NC, USA).

### Ethics approval and consent to participate

The KLoSA study was approved by the National Statistical Office (Approval number: 33602) and was conducted after acquiring verbal consent from participants in the study. Since the KLoSA database has been released to the public for scientific use, ethical approval was not required for this study.

### Consent for publication

There are no details of individual participants in the manuscript.

## Supplementary Information

Supplementary Information.

## Data Availability

The data used in this study are available at https://survey.keis.or.kr/eng/klosa/databoard/List.jsp.
