# Supplementary material for: Frailty transitions and cognitive function among South Korean older adults
Source: Sci Rep. 2021 May 20;11:10658. doi: 10.1038/s41598-021-90125-6 (PMC8138002; doi:10.1038/s41598-021-90125-6)
Supplement: Supplementary file 1 — Supplementary Information. [file 41598_2021_90125_MOESM1_ESM.docx]

| **Supplementary table 1. Association of Frailty Instrument (FI) transitions with cognitive function** | | | | | | | | |
| --- | --- | --- | --- | --- | --- | --- | --- | --- |
| **Variables** | | **Cognitive function (K-MMSE)** | | | | | | |
|  |  | **Men** | | |  | **Women** | | |
|  |  | **ß** | **SE** | **P-value** |  | **ß** | **SE** | **P-value** |
| **Changes in FI** | |  |  |  |  |  |  |  |
| Robust→Robust |  | **Ref.** |  |  |  | **Ref.** |  |  |
| Robust→Prefrail |  | -0.309 | 0.148 | 0.0362 |  | -1.184 | 0.181 | <.0001 |
| Robust→Frail |  | -1.850 | 0.341 | <.0001 |  | -2.784 | 0.368 | <.0001 |
| Prefrail→Prefrail |  | -1.061 | 0.163 | <.0001 |  | -0.836 | 0.160 | <.0001 |
| Prefrail→Robust |  | 0.057 | 0.152 | 0.7089 |  | -0.273 | 0.185 | 0.1399 |
| Prefrail→Frail |  | -2.092 | 0.289 | <.0001 |  | -2.170 | 0.263 | <.0001 |
| Frail→Frail |  | -2.402 | 0.268 | <.0001 |  | -2.392 | 0.232 | <.0001 |
| Frail→Prefrail |  | -1.132 | 0.302 | 0.0002 |  | -1.016 | 0.259 | <.0001 |
| Frail→Robust |  | -0.722 | 0.470 | 0.1244 |  | -0.511 | 0.383 | 0.1822 |
| **K-MMSE, Korean-Mini Mental Status Evaluation; β, regression coefficient; SE, standard error.** | | | | | | | | |
| ***Adjusted for other covariates** | | | | | | | | |

| **Supplementary table 2. Differences in frailty score changes by wave** | | | | | | | |
| --- | --- | --- | --- | --- | --- | --- | --- |
| **Variables** | | | **Men** | |  | **Women** | |
|  |  |  | N | % |  | N | % |
| **Frailty score changes^†^(2008→ 2010)** | | | |  |  |  |  |
| -3 |  |  | 2 | (0.2) |  | 4 | (0.3) |
| -2 |  |  | 26 | (2.4) |  | 55 | (4.3) |
| -1 |  |  | 180 | (16.3) |  | 210 | (16.5) |
| 0 |  |  | 564 | (51.2) |  | 632 | (49.6) |
| 1 |  |  | 276 | (25.1) |  | 293 | (23.0) |
| 2 |  |  | 49 | (4.5) |  | 75 | (5.9) |
| 3 |  |  | 4 | (0.4) |  | 5 | (0.4) |
| **Total** |  |  | 1,101 | (100.0) |  | 1,274 | (100.0) |
| **Frailty score changes^†^(2010→ 2012)** | | | |  |  |  |  |
| -3 |  |  | 0 | (0) |  | 4 | (0.4) |
| -2 |  |  | 15 | (1.6) |  | 32 | (2.8) |
| -1 |  |  | 173 | (18.3) |  | 235 | (20.9) |
| 0 |  |  | 517 | (54.7) |  | 597 | (53.0) |
| 1 |  |  | 215 | (22.8) |  | 219 | (19.4) |
| 2 |  |  | 23 | (2.4) |  | 37 | (3.3) |
| 3 |  |  | 2 | (0.2) |  | 2 | (0.2) |
| **Total** |  |  | 945 | (100.0) |  | 1,126 | (100.0) |
| **Frailty score changes^†^(2012→ 2014)** | | | |  |  |  |  |
| -3 |  |  | 2 | (0.2) |  | 3 | (0.3) |
| -2 |  |  | 23 | (2.8) |  | 35 | (3.6) |
| -1 |  |  | 161 | (19.4) |  | 182 | (18.8) |
| 0 |  |  | 419 | (50.5) |  | 487 | (50.3) |
| 1 |  |  | 187 | (22.5) |  | 211 | (21.8) |
| 2 |  |  | 35 | (4.2) |  | 47 | (4.9) |
| 3 |  |  | 3 | (0.4) |  | 3 | (0.3) |
| **Total** |  |  | 830 | (100.0) |  | 968 | (100.0) |
| **Frailty score changes^†^(2014→ 2016)** | | | |  |  |  |  |
| -3 |  |  | 0 | (0) |  | 1 | (0.1) |
| -2 |  |  | 13 | (1.8) |  | 25 | (3.0) |
| -1 |  |  | 127 | (17.7) |  | 189 | (22.3) |
| 0 |  |  | 390 | (54.4) |  | 429 | (50.6) |
| 1 |  |  | 151 | (21.1) |  | 170 | (20.1) |
| 2 |  |  | 26 | (3.6) |  | 30 | (3.5) |
| 3 |  |  | 10 | (1.4) |  | 3 | (0.4) |
| **Total** |  |  | 717 | (100.0) |  | 847 | (100.0) |
| **Frailty score changes^†^(2016→ 2018)** | | | |  |  |  |  |
| -3 |  |  | 1 | (0.2) |  | 0 | (0) |
| -2 |  |  | 15 | (2.5) |  | 26 | (3.5) |
| -1 |  |  | 101 | (16.7) |  | 148 | (19.8) |
| 0 |  |  | 313 | (51.9) |  | 367 | (49.2) |
| 1 |  |  | 136 | (22.6) |  | 169 | (22.7) |
| 2 |  |  | 36 | (6.0) |  | 33 | (4.4) |
| 3 |  |  | 1 | (0.2) |  | 3 | (0.4) |
| **Total** |  |  | 603 | (100.0) |  | 746 | (100.0) |
| **K-MMSE, Korean-Mini Mental Status Evaluation; β, regression coefficient; SE, standard error** | | | | | | | |
| ***Adjusted for other covariates** | | | | | | | |
| **† Indicates differences in frailty score changes from preceding year to following year** | | | | | | | |
